# Supplementary material for: Continuous artificial synthesis of glucose precursor using enzyme-immobilized microfluidic reactors
Source: Nat Commun. 2019 Sep 6;10:4049. doi: 10.1038/s41467-019-12089-6 (PMC6731257; doi:10.1038/s41467-019-12089-6)
Supplement: Supplementary file 1 — Supplementary Information [file 41467_2019_12089_MOESM1_ESM.pdf]

Supplementary Information

Continuous artificial synthesis of glucose precursor using enzyme-immobilized microfluidic reactors

Zhu et al.

Supplementary Methods

Modification of pristine PDMS microfluidic reactors with PDA

Briefly, the PDMS microfluidic reactor was first cleaned by 1 M NaOH solution, and then infused with 10% (v/v) APTES solution. The reactor was incubated at 50 ºC for 3 hours for amine-functionalization. The APTES-treated microfluidic reactor was further rinsed with ethanol, dried, and baked at 125 ºC for 1 hour. Then, 1.5 mL dopamine solution (1 mg·mL^{-1}) was injected into the microfluidic reactor under the flow rate of 2.5 μL·min^{-1} on a hot plate at 50 ºC. After being rinsed with 10 mM Tris-HCl buffer (pH 8.8), the microfluidic reactor was successfully modified with PDA and was ready for the enzyme immobilization.

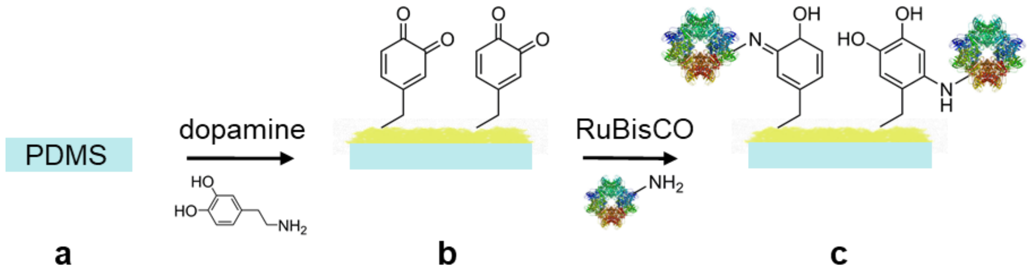

Supplementary Figure 1. Illustration of the procedures of RuBisCO immobilization on PDMS by PDA. (a) Pristine PDMS. (b) PDA-modified PDMS. (c) RuBisCO-immobilized PDMS. The amine binding to the aromatic ring at the para-position of hydroxyl here is a possible structure for the bonding of RuBisCO to PDA by Michael addition[1].

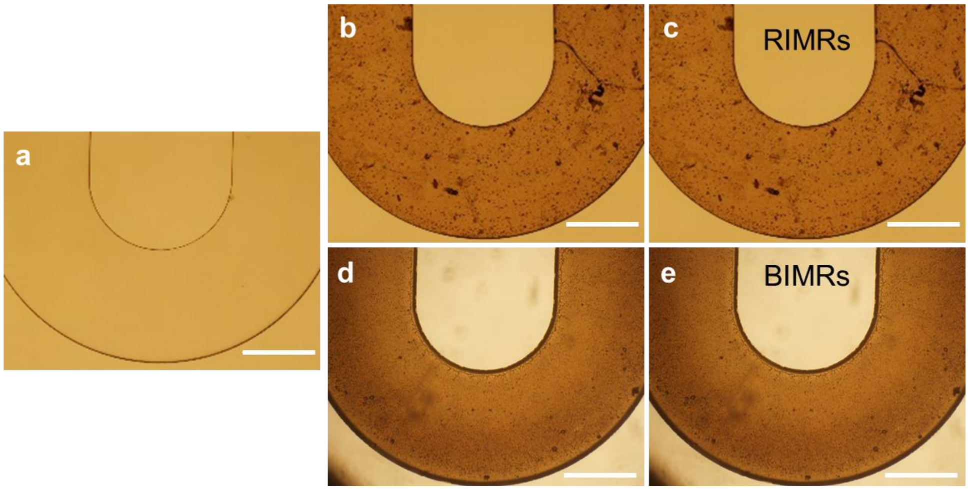

Supplementary Figure 2. Optical microscopic images of microchannels after each step of the immobilization procedures. (a) is the pristine PDMS microchannel, which is transparent. (b) and (d) are PDA-modified microchannels, which become opaque and brown. (c) is the RuBisCO-immobilized microchannel and (e) is the BSA-immobilized microchannel. The subsequent RuBisCO immobilization or BSA immobilization would not change the surface roughness. The images are obtained from Olympus BX41. The scale bar is 500 μm.

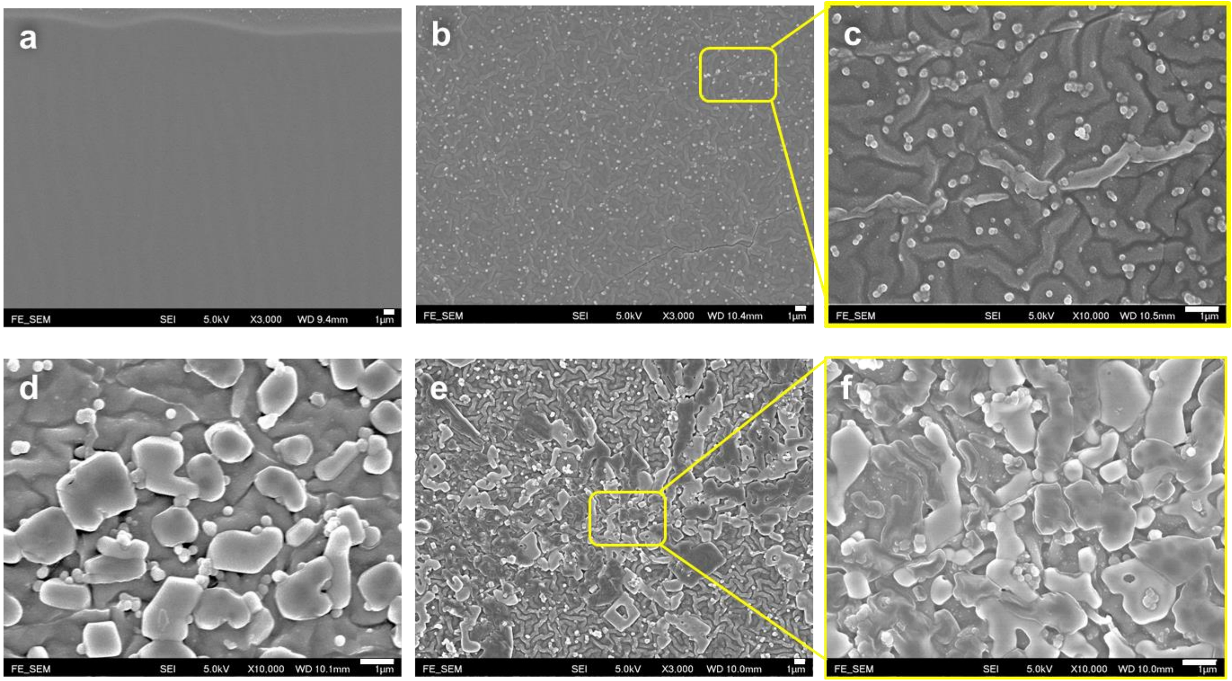

Supplementary Figure 3. SEM images for the surface characterization of the microchannels' inner surfaces. (a) is the pristine PDMS surface, which is flat and smooth. (b) and (c) are the PDA-modified PDMS surfaces. After the PDA modification, a rough layer with many nanoparticles is formed. This coarse PDA layer can provide a much larger surface area than the smooth PDMS surface, offering more active functional groups to covalently couple with RuBisCO. (d) is the RuBisCO-immobilized PDA-PDMS surface at the magnification of 10000×. The immobilization of RuBisCO brings about many blocks onto the PDA layer. (e) and (f) are the BSA-immobilized PDA-PDMS surface. The blocks formation can also be observed when BSA is immobilized into the microreactors. The SEM images obtained from JEOL JSM-6490. The scale bar is 1 μm.

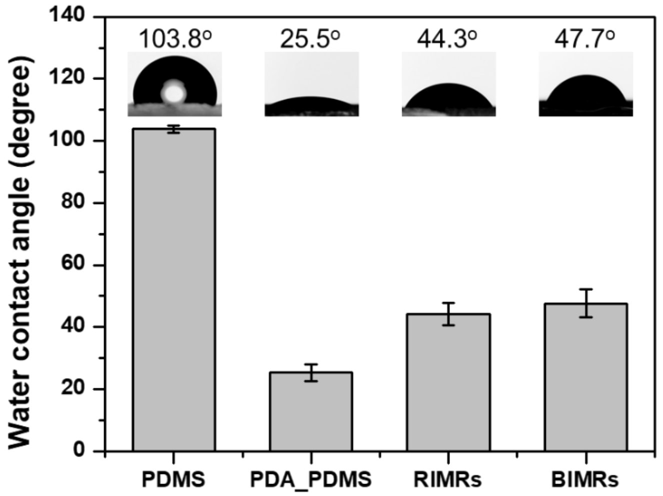

Supplementary Figure 4. Water contact angles of PDMS, PDA-modified PDMS, RIMRs and BIMRs surfaces. The water contact angle of the inner surface significantly decreases from 103.8° to 25.5° after the PDA modification. This hydrophilicity improvement results from the abundant catechol groups of PDA. In contrast, further immobilization of RuBisCO leads to an increase of water contact angle, which is probably due to the hydrophobic side chains of amino acids in RuBisCO. For the control experiments, the BSA immobilization also presents similar results. Water contact angles results are conducted by a standard contact angle goniometer (Model 200, ramé-hart instrument co.). Error bars represent the standard deviations from three independent experiments. Source data are provided as a Source Data file.

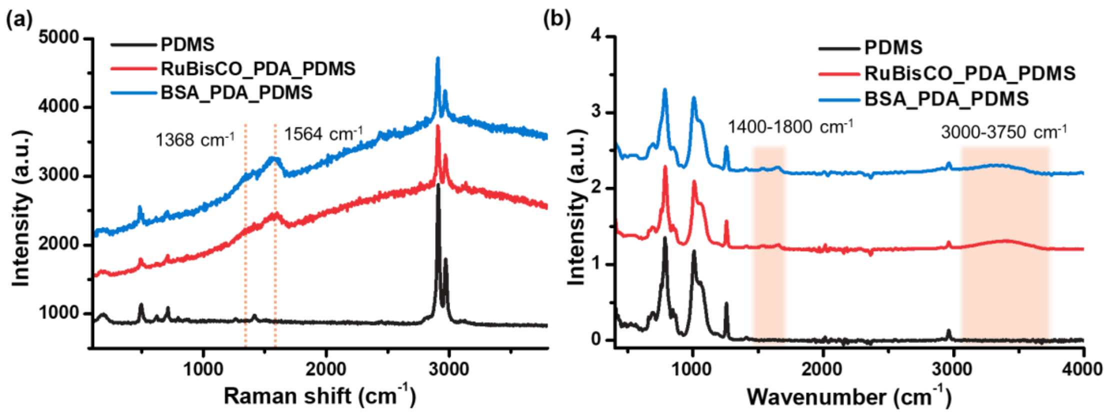

Supplementary Figure 5. Raman and ATR-FTIR spectra of microchannels. (a) are the Raman spectra obtained by Witec_Confocal Raman system. (b) are the ATR-IR spectra obtained by an attenuated total reflection Fourier transform infrared spectroscopy (ATR-FTIR, BRUKER). New peaks (1368 cm^{-1} and 1564 cm^{-1} in Raman spectra and 1400-1800 cm^{-1} and 3000-3750 cm^{-1} in ATR-FTIR spectra) can be observed after the PDA modification and the RuBisCO/BSA immobilization when compared with the pristine PDMS microfluidic channels. Particularly, the FTIR spectrum of the RIMRs has a weak band at about 1600 cm^{-1}, which could be referred to the vibrational modes of the peptide bond (amide bands). This absorption band confirms the effective RuBisCO immobilization and gives information about conformational changes induced by the immobilization procedure. Source data are provided as a Source Data file.

Fluorescence experiment

The fluorescence experiment was also conducted to verify the RuBisCO immobilization. The RuBisCO-FITC (RuBisCO tagged by fluorescein isothiocyanate) solution was introduced into the PDA-PDMS microfluidic reactor and then kept at room temperature for 6 hours. The reactor was observed under a fluorescence microscope (Olympus BX41) to record the fluorescence images. Afterwards, it was thoroughly rinsed by 0.1-M phosphate-buffered saline (PBS, pH 9.2) to check whether the fluorescence emission still existed.

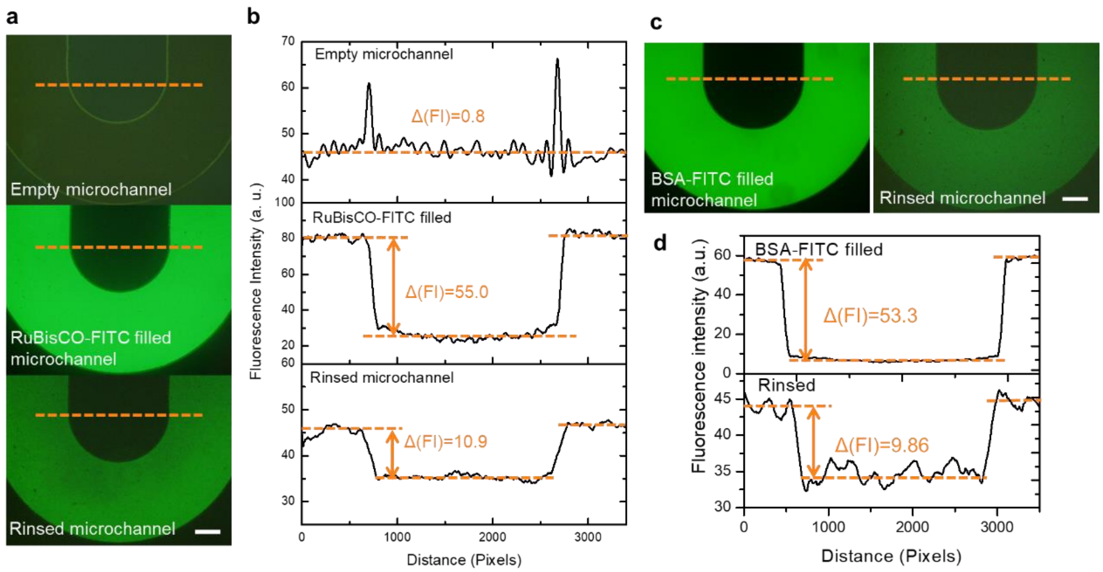

Supplementary Figure 6. Fluorescence experiments for confirming RuBisCO immobilization. (a) Fluorescence images of the empty microchannel, the RuBisCO-FITC filled microchannel, and the rinsed microchannel. (b) The corresponding fluorescence intensity profiles obtained along the observation lines. (c) Fluorescence images and (d) fluorescence intensity profiles of the BSA-FITC filled microchannel and the rinsed microchannel. The fluorescence intensity difference (noted as Δ(FI)) between the microchannel and the microchannel wall is significantly increased after the injection of the RuBisCO-FITC solution. After the thoroughly rinsing by the PBS buffer, Δ(FI) decreases but not drops to zero. The non-zero Δ(FI) after the rinse proves that a portion of RuBisCO is well retained on the microchannel surface and the strategy of RuBisCO immobilization on microfluidic reactor via PDA modification is feasible. Similar results are also observed in the BSA-immobilized microfluidic reactors. The scale bar is 500 μm. Source data are provided as a Source Data file.

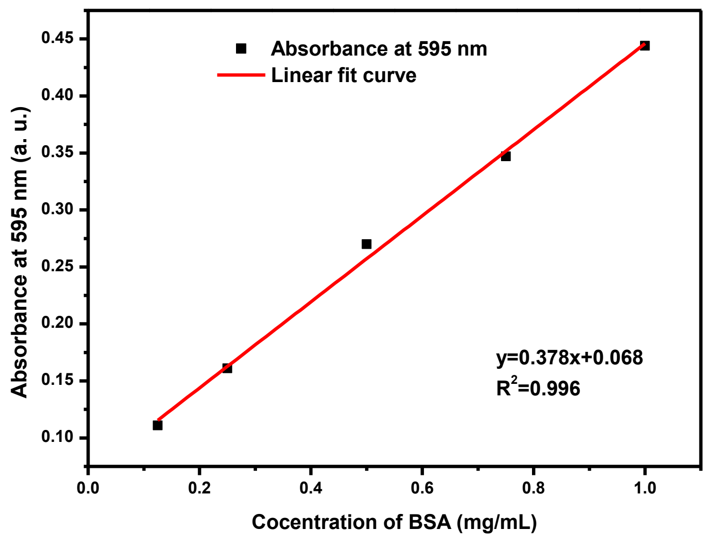

Supplementary Figure 7. Calibration of protein amount determined from BSA solutions by the Bradford method. Protein amount was qualified using the Quick Start Bradford Protein Assay kit (Bio-Rad Pacific Limited.), which were determined by measuring the absorbance at the wavelength of 595 nm using a UV-Visible spectrometer (UV-2450, Shimadzu). BSA solutions (0.125-1 mg·mL^{-1}) were selected as standards to plot the calibration curve. Source data are provided as a Source Data file.

Amplification method for RuBisCO activity assay

For the immobilized RuBisCO activity assay, reactant mixture (66 mM HCO_3^- and 0.5 mM RuBP in the reaction buffer) was passed through the RIMRs and the production solution I (containing RuBisCO, RuBP, HCO_3^- and 3-PGA in the reaction buffer) was collected from the outlet of the reactors for further assay. In terms of the free RuBisCO activity assay, RuBisCO was incubated with reactant mixture for 1 min before the reaction was stopped with the same volume of 80% ethanol. The production solution II (containing RuBisCO, RuBP, HCO_3^-, products and ethanol in the reaction buffer) was kept for further assay. The amount of RuBisCO used here was the same as that immobilized into the microfluidic reactors (21.875 μg). 20 μL of the production solutions (i.e., production solution I for immobilized RuBisCO assay and production solution II for free RuBisCO assay) were added with 80 μL of assay mixture (the final concentrations were 5 unit·mL^{-1} PGK, 0.5 unit·mL^{-1} GAPDH, 0.5 unit·mL^{-1} TPI, 0.5 unit·mL^{-1} G3PDH, 1 unit·mL^{-1} G3POX, 1000 unit·mL^{-1} catalase, 0.5 mM ATP, 2 mM NADH, 1.5 mM $MgCl_2$ and 100 mM Tricine/KOH pH 8.0). The reaction was immediately and continuously monitored by measuring the absorbance change at 340 nm by a UV-Visible spectrometer. During the reaction, the product 3-PGA was first converted to dihydroxyacetone-phosphate (DAP) with PGK, GAPDH, ATP and NADH. Catalase was also added here to prevent the inhibition of GAPDH. Then, DAP was transformed into the cycle of mutual conversion with glycerol-3 phosphate (G3P). It can be monitored as the cumulative oxidation of NADH, whose amount is much larger than the original amount of 3-PGA, therefore providing strong amplification of signal for 3-PGA monitoring (Supplementary Figure 8a). The signal of production solutions can be converted to the specific amount of 3-PGA by using a standard curve generated by adding different amounts of standard 3-PGA into the assay mixture as shown by the dark solid squares in Supplementary Figure 8b. Calibration for standard 3-PGA

in the mixture of reaction buffer:ethanol (60:40, v/v) are also plotted by the red open circles in Supplementary Figure 8b to determine the 3-PGA amount in production solution II.

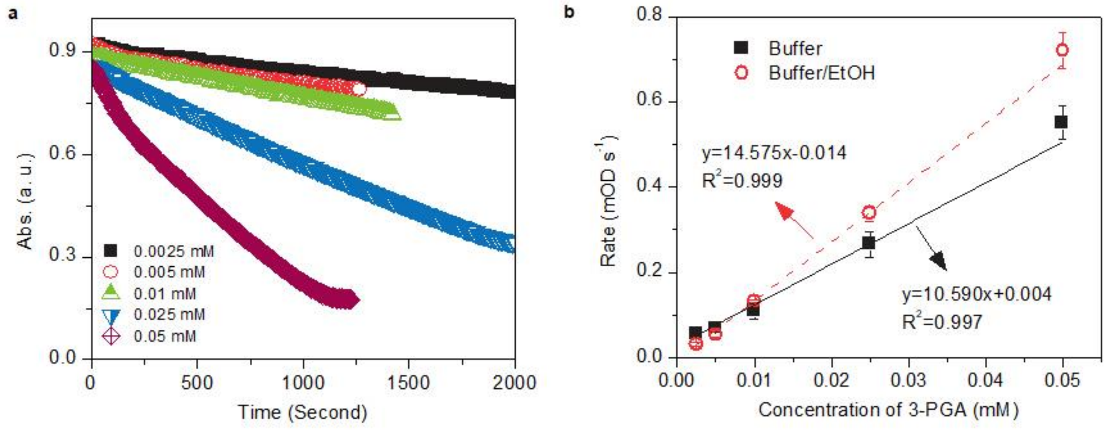

Supplementary Figure 8. Calibration of standard 3-PGA amount by UV-Visible spectrometer. (a) Decrease of the absorbance at 340 nm as a function of the time for different concentrations (from 0.0025 to 0.05 M) of 3-PGA dissolved in the reaction buffer. (b) Calibration curves of 3-PGA both in the reaction buffer and in the mixture of reaction buffer:ethanol (60:40, v/v) mixture by the amplification signal assay with using a UV-Vis spectrometer. Error bars represent the standard deviations from three independent experiments. Source data are provided as a Source Data file.

Determination of the reaction time in RIMRs

The reaction time t_r is regarded as the residence time of the reaction mixture flowing through the RIMRs, which is calculated by the equation of $t_r = V_r / Q$, where V_r is the volume of the RIMRs and Q is the flow rate of the injected RuBP solution controlled by the syringe pump. In this work, the volume of RIMR is 7 μL, the corresponding reaction time is 1 min, 5 min, 7 min, 10 min for the flow rates of 7 μL·min^{-1}, 1.4 μL·min^{-1}, 1 μL·min^{-1}, 0.7 μL·min^{-1}, respectively.

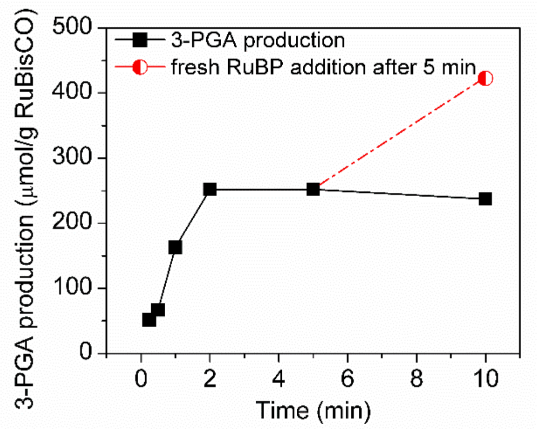

Supplementary Figure 9. Production of 3-PGA by free RuBisCO as a function of time. For the 3-PGA production with the reaction time of 10 min, two tests are conducted to explore the reason of no 3-PGA producing after 2 min-reaction. One test directly uses the production solution I after 10 min for analysis. As shown by dark squares, the production of 3-PGA still does not increase. The other one is to add fresh RuBP into the production solution I after 5 min (final RuBP concentration is 0.5 mM and HCO_3^- is 66 mM) and then to collect the production solution I after another 5 min for analysis (red circles). By contrast, more 3-PGA is produced this time, which shows that the stopped increasing of 3-PGA after 2 min is due to the exhaustion of RuBP. The amount of RuBisCO used is 21.875 μg. Production solution I is collected for 21 μL. Source data are provided as a Source Data file.

HPLC-MS/MS analysis

The monitoring of the reaction was performed using a liquid chromatography-tandem mass spectrometer (LC-MS/MS) system that consisted of Agilent 1290 Infinity LC and 6460 Electrospray Ionization Triple Quadrupole Mass Spectrometer (Agilent Technologies, Palo Alto, CA, USA). A Luna NH_2 column (Phenomenex, 2mm×150mm, 5μm) was used for the chromatography separation. The mobile phases consisted of (A) 20 mM ammonium acetate and 20 mM ammonium hydroxide in water (pH 9.45), and (B) acetonitrile (ACN):water (85:15, v/v). LC separation was carried out at 25°C with a linear gradient elution condition at a flow rate of 0.4

mL·min^{-1} with an injection volume of 5 μL. The LC conditions were as follows: gradient elution from 100% to 0% B in 15 min (held for 5 min), then went back to 100% B in 2 min and held for 10 min until the column reached equilibrium.

The MS/MS system was equipped with an electrospray ionization source operated under negative mode. The multi-reaction monitoring (MRM) was used in the MS detection with the transitions (309→97) and (185→97) for RuBP and 3-PGA, respectively. All the samples were dissolved in the mixture of reaction buffer:ACN (50:50, v/v).

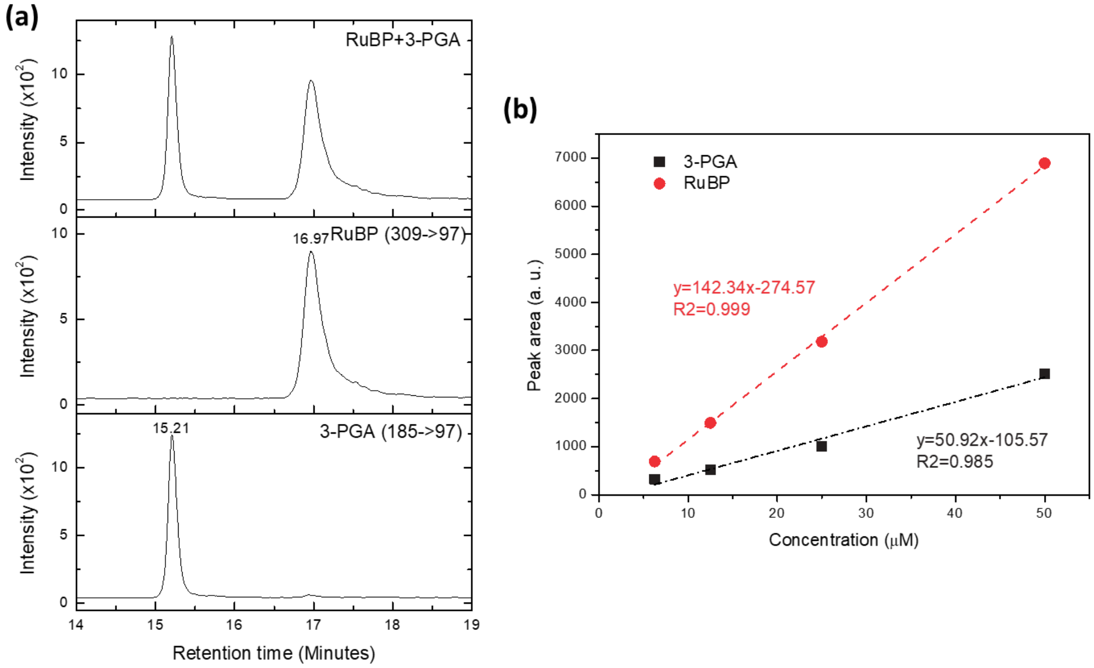

Supplementary Figure 10. HPLC-MS/MS analysis of the standard RuBP and 3-PGA. (a) HPLC-MS/MS chromatography of the standard RuBP and 3-PGA with the concentration of 100 μM. The retention times of RuBP and 3-PGA were 16.97 min and 15.21 min, respectively. (b) Calibration of the peak areas of the RuBP and 3-PGA chromatography as a function of their concentrations. The coefficients of determination for both RuBP and 3-PGA are only acceptable when the concentration is smaller than 50 μM. Source data are provided as a Source Data file.

Supplementary Figures

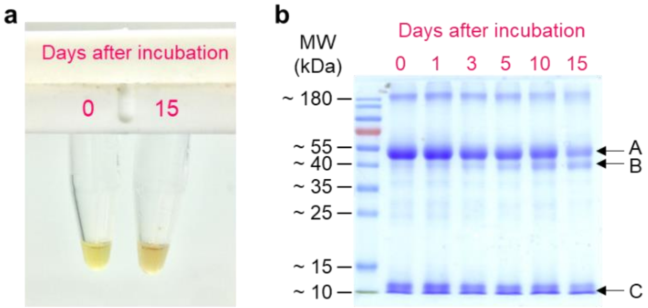

Supplementary Figure 11. Characterization of free RuBisCO for different incubation time. (a) Photograph of RuBisCO incubated in the reaction buffer at 4 °C for 0 day and 15 days. No protein participants were observed after long time storage. However, the solution color changed from light yellow to brownish yellow. (b) SDS-PAGE analysis of RuBisCO incubated in reaction buffer at 4 °C for different days. 40 μL of 5-μg·μL^{-1} RuBisCO in the reaction buffer incubated for different days were added to 25 μL of SDS dye and then boiled for 10 min. Next, 8 μL of the mixtures were loaded to SDS-PAGE (7% SDS gel). The most significant protein bands are detected at ~ 54 kDa (A) and ~ 13 kDa (C) which correspond to large subunits (LSU) and small subunits (SSU) of RuBisCO, respectively. The color of protein band of LSU tends to be lighter with the increase of incubation time, and that of RuBisCO incubated for 15 days is the lightest. After days of incubation, an extra protein band appears at ~ 40 kDa (B), which may be the break-down product of LSU[2,3]. This explains that the protein degradation may be responsible for the activity drop of free RuBisCO after incubation. Each lane contains about 24.6 μg of RuBisCO. Staining was carried out with Coomassie Blue stain. Source data are provided as a Source Data file.

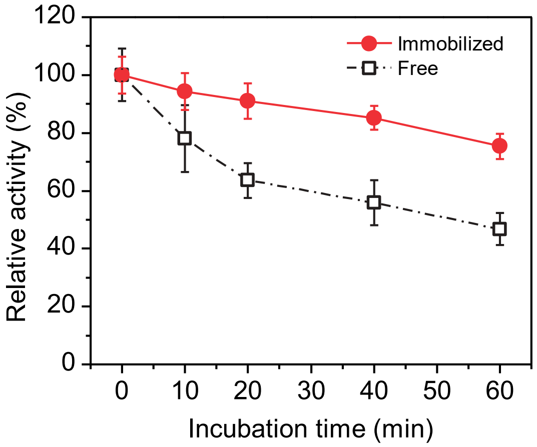

Supplementary Figure 12. Relative activities of RuBisCO retained after a prolonged incubation time up to 60 min at 50 °C. Dark open squares represent the relative activity of the free RuBisCO and Red solid circles represent that of the immobilized RuBisCO. The amount of RuBisCO used is 21.875 μg for both the immobilized and free ones. RuBP concentration is 0.5 mM and HCO_3^- is 66 mM. The collected production solutions are 100 μL. Error bars represent the standard deviations from three independent experiments. Source data are provided as a Source Data file.

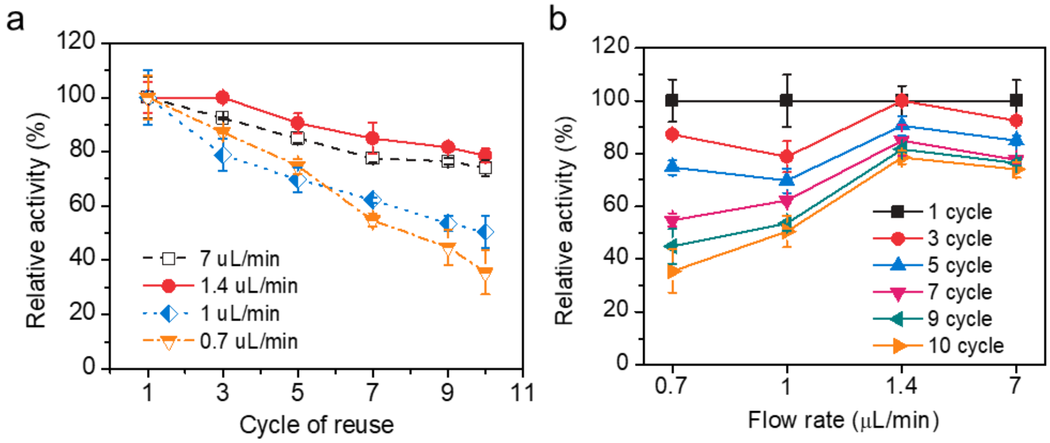

Supplementary Figure 13. Reusability of the RIMRs when the reactant mixture is injected at different flow rates. The flow rates are set as 7 μL·min^{-1}, 1.4 μL·min^{-1}, 1 μL·min^{-1} and 0.7 μL·min^{-1}. As the volume of reactor is 7 μL, the corresponding reaction time is 1 min, 5 min, 7 min and 10 min, respectively. (a) Reusability as a function of cycle of reuse at different flow rates. The cycles of reuse are up to ten. (b) Reusability as a function of flow rate after different cycles of reuse. RuBP concentration is 0.5 mM and HCO_3^- is 66 mM. Error bars represent the standard deviations from three independent experiments. Source data are provided as a Source Data file.

Supplementary References

1. Chao, C. et al. Surface modification of halloysite nanotubes with dopamine for enzyme immobilization. *ACS Appl. Mater. Interfaces 5*, 10559-10564, (2013).
2. Zhu, W. Y. *et al.* Evidence of a Role for Plant Proteases in the Degradation of Herbage Proteins in the Rumen of Grazing Cattle. *J. Dairy Sci.* **82**, 2651-2658, (1999).
3. Kokubun, N., Ishida, H., Makino, A. & Mae, T. The Degradation of the Large Subunit of Ribulose-1,5-bisphosphate Carboxylase/oxygenase into the 44-kDa Fragment in the Lysates of Chloroplasts Incubated in Darkness. *Plant Cell Physiol.* **43**, 1390-1395, (2002).
